# A reference library for Canadian invertebrates with 1.5 million barcodes, voucher specimens, and DNA samples

**DOI:** 10.1038/s41597-019-0320-2

**Published:** 2019-12-06

**Authors:** Jeremy R. deWaard, Sujeevan Ratnasingham, Evgeny V. Zakharov, Alex V. Borisenko, Dirk Steinke, Angela C. Telfer, Kate H. J. Perez, Jayme E. Sones, Monica R. Young, Valerie Levesque-Beaudin, Crystal N. Sobel, Arusyak Abrahamyan, Kyrylo Bessonov, Gergin Blagoev, Stephanie L. deWaard, Chris Ho, Natalia V. Ivanova, Kara K. S. Layton, Liuqiong Lu, Ramya Manjunath, Jaclyn T. A. McKeown, Megan A. Milton, Renee Miskie, Norm Monkhouse, Suresh Naik, Nadya Nikolova, Mikko Pentinsaari, Sean W. J. Prosser, Adriana E. Radulovici, Claudia Steinke, Connor P. Warne, Paul D. N. Hebert

**Affiliations:** 1 Centre for Biodiversity Genomics, University of Guelph, Guelph, Ontario, Canada; 2 Present Address: Public Health Agency of Canada, Guelph, Ontario Canada; 3 Present Address: Ocean Frontier Institute, Dalhousie University, Halifax, Nova Scotia Canada

**Keywords:** Biodiversity, Taxonomy, Molecular ecology, Next-generation sequencing, Entomology

## Abstract

The reliable taxonomic identification of organisms through DNA sequence data requires a well parameterized library of curated reference sequences. However, it is estimated that just 15% of described animal species are represented in public sequence repositories. To begin to address this deficiency, we provide DNA barcodes for 1,500,003 animal specimens collected from 23 terrestrial and aquatic ecozones at sites across Canada, a nation that comprises 7% of the planet’s land surface. In total, 14 phyla, 43 classes, 163 orders, 1123 families, 6186 genera, and 64,264 Barcode Index Numbers (BINs; a proxy for species) are represented. Species-level taxonomy was available for 38% of the specimens, but higher proportions were assigned to a genus (69.5%) and a family (99.9%). Voucher specimens and DNA extracts are archived at the Centre for Biodiversity Genomics where they are available for further research. The corresponding sequence and taxonomic data can be accessed through the Barcode of Life Data System, GenBank, the Global Biodiversity Information Facility, and the Global Genome Biodiversity Network Data Portal.

## Abstract (summary)

**Table:** 

Measurement(s)	DNA • digital imaging
Technology Type(s)	taxonomic diversity assessment by targeted gene survey • digital curation
Factor Type(s)	species • geographic location • habitat
Sample Characteristic - Organism	Metazoa
Sample Characteristic - Environment	terrestrial biome • freshwater biome • marine biome
Sample Characteristic - Location	Canada

Machine-accessible metadata file describing the reported data: 10.6084/m9.figshare.10298354

## Background & Summary

High-throughput sequencing platforms have enabled a novel approach to biodiversity surveys by making it possible to identify the array of species present in bulk collections or environmental DNA^1–3^. This ‘metabarcoding’ approach is enabling rapid assessment of the species composition of complex substrates, communities, and environments, from gut contents and feces to soil and aquatic systems, to ancient environments captured in sediments or permafrost^1,2,4,5^. The process begins by obtaining large numbers of sequence records from one or more target amplicons, or DNA barcodes^6^ from the constituent organisms. This is followed by querying these sequences against a reference library derived from carefully identified records. Two reference databases are commonly employed for macro-organisms: NCBI’s GenBank^7^ and the Barcode of Life Data System (BOLD^8^). Custom reference libraries are more restrictive in taxonomic scope, but they are less prone to introducing errors in identification (e.g.^9,10^).

An adequately parameterized reference library is critical for robust taxonomic assignments (e.g.^11,12^). These libraries gain utility and reliability when each species is represented by multiple, geographically distinct populations^13,14^ to capture maximal intraspecific variation and in turn, define the boundaries of and between species. As well, particularly for speciose genera, the libraries should ideally include multiple species^15^. The use of authoritatively identified records can provide reliable species-level assignments, but can also reveal limitations of the barcode marker(s) in particular taxa^11,13,16,17^. A significant proportion of the barcode records in reference databases are only identified to a family level^17,18^ in part because some 90% of multicellular species are undescribed. Despite the lack of species-level identifications, such records are useful for assigning taxa to higher taxonomic categories^8,12,14^. While libraries with comprehensive species coverage are the ultimate goal (e.g.^19^), it is estimated that only 15% of described animal species are currently represented in public databases^20^. There is, however, a strong prospect that coverage will rise rapidly with the introduction of high throughput sequencing (HTS) protocols which come with lower analytical costs^21–23^, and can recover sequences from museum and type specimens^24^.

While the reliability of identifications generated through metabarcoding studies depends mainly upon access to a validated reference library constructed with expert taxonomic knowledge, the quality of the libraries is reinforced by access to the voucher specimens, to high-quality images of these specimens, and to genomic DNA samples from them. The importance of retaining voucher specimens in systematic and ecological studies is well-appreciated^25–28^ – they allow future taxonomic examination which, in turn, can prevent the proliferation of incorrect identifications or ‘error cascades’^29^. Because the retention of specimens used as a source for DNA has not been required for sequences submitted to GenBank, it contains many sequences derived from specimens which were discarded, destroyed, or misidentified^27^. Moreover, corrections to sequences assigned to the wrong species in GenBank can only be made by the data submitter, a serious impediment to curation^30^. Curation of reference libraries is also facilitated by high-resolution images of specimens, either as ‘e-vouchers’^31^ that can be digitally distributed, or better yet, images are taken in addition to voucher specimens. It is also apparent that cryopreserving genomic DNA extracts derived from the construction of reference libraries will become increasingly important^32,33^. DNA vouchering provides another opportunity for taxonomic ‘quality control’, in those taxa where the primary barcode region fails to differentiate closely-related taxa, as additional sequence information can be gathered (e.g. 16S rRNA^5^). In fact, the retention of DNA extracts provides a basis for ‘upgrading’ records if the barcode standard eventually adopts new approaches in groups, such as plants, where data standards have changed^34^, or for sequencing the entire genome^35^.

This data resource presents a curated DNA barcode reference library for a sizeable fraction of the Canadian invertebrate fauna, illustrates the workflows involved in its construction, and describes the resources resulting from this effort. The library was constructed and curated for over a decade, beginning with collecting efforts at sites across Canada, followed by specimen processing and DNA barcoding, and finally, taxonomic identification and validation. The library includes barcode records for 1.5 million specimens representing nearly 65,000 species, all supported by voucher specimens, digital images, and DNA extracts available for follow-up studies. Despite a national focus, we anticipate it will have wide utility in metabarcoding and related studies, due to the diversity of taxa included.

## Methods

**Fig. 1:**
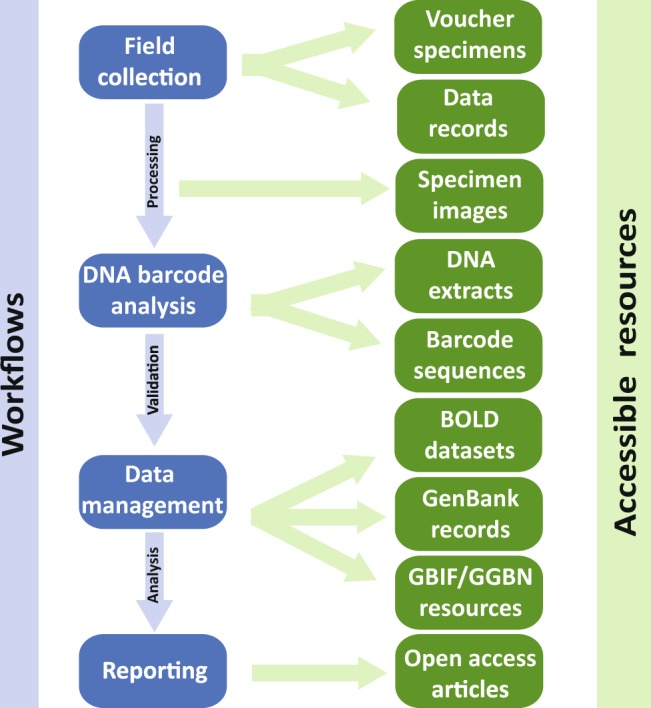
Overview of the study design to create and maintain the curated reference DNA barcode library for Canadian invertebrates.

The curated DNA barcode reference library presented here for Canadian invertebrates was constructed by a series of workflows that generate diverse products and accessible resources (Fig. 1).

### Field collections

All collecting was done in compliance with national and provincial regulations and appropriate permits were obtained where necessary. No permits are required to collect invertebrates from public areas in Canada, except for species at risk; none of the collecting efforts targeted endangered or protected species. Collections of invertebrates within Canadian National Parks were made under permits NAP-2008–1636 (2008–2010), PC-2012-11074 (2012–2014), and NAP-2015-19000 (2015–2017) granted by Parks Canada. Permits and/or permissions were also obtained for invertebrate sampling in provincial parks (e.g. BC provincial parks in 2014 – British Columbia Ministry of Environment # 107242), municipal parks, conservation authorities, research properties, and other protected areas (e.g. Nature Conservancy of Canada properties).

### Sampling localities

**Fig. 2:**
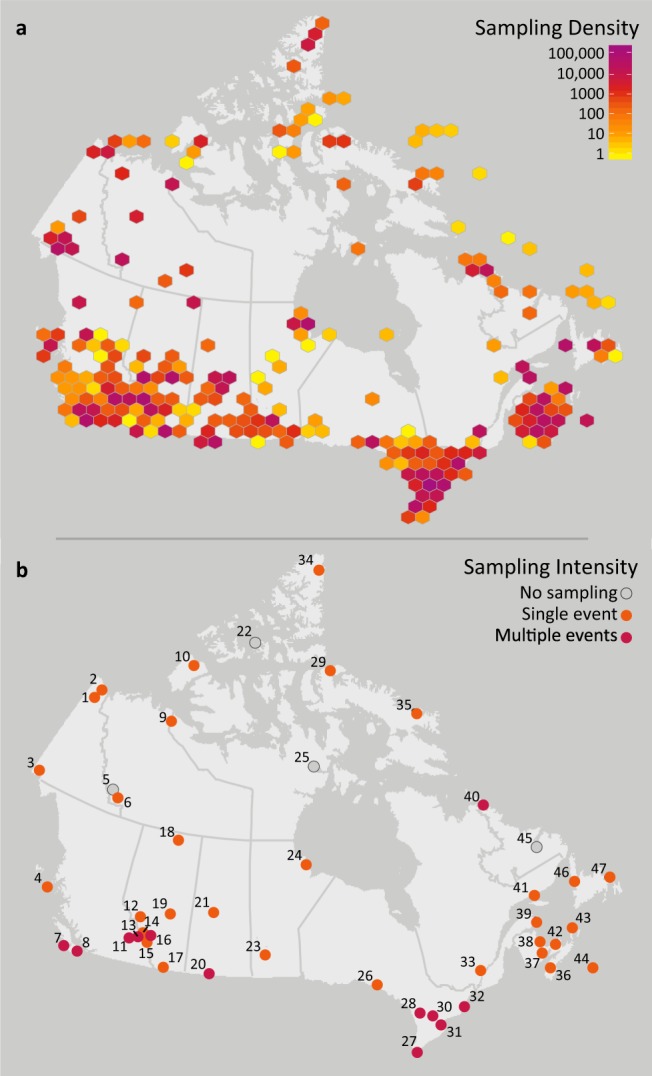
Geographic coverage of the Canadian specimen data release. (**a**) The sampling density for the complete dataset of 1,500,003 specimen records. (**b**) The sampling intensity for the 47 National Parks, National Park Reserves, and National Urban Parks of Canada. Numbers correspond to Parks in Online-only Table 1.

**Fig. 3:**
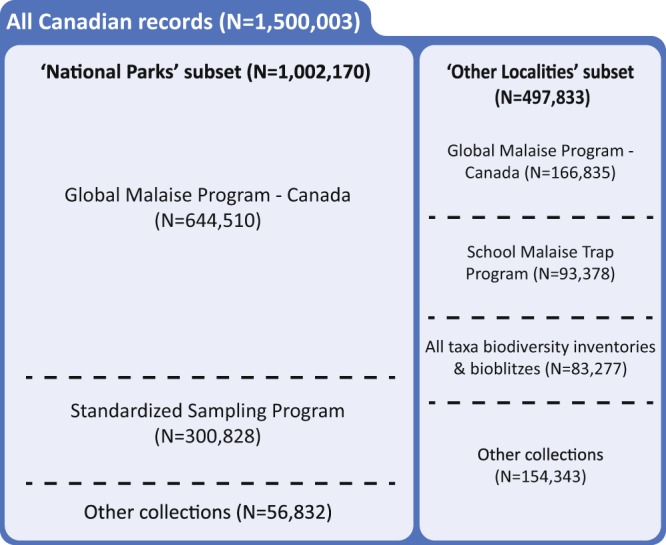
Breakdown of the Canadian specimen data release by sampling program. N = number of records.

Specimens were collected from 3413 locations (Fig. 2a), representing all 13 provinces and territories of Canada, and all 23 of its terrestrial and aquatic ecozones^36^. Collecting sites included national parks, provincial parks, municipal parks, conservation reserves, research reserves, other protected areas, school grounds, as well as industrial and residential properties. Of the 47 National Parks, National Park Reserves, and National Urban Parks in Canada, collecting was undertaken in 43 (Fig. 2b). In total, 1,500,003 specimens were collected and underwent DNA barcoding (see below), of which 1,002,170 were collected within national park boundaries (herein, the ‘National Parks’ subset) and 497,833 elsewhere (the ‘Other Localities’ subset) (Fig. 3).

### Sampling methods and programs

The 1,500,003 specimens included in this data release were obtained by both active and passive collecting methods, and as part of various sampling programs across Canada (Fig. 2a, details below; Fig. 2b). Specimens were predominantly collected between 2008 and 2017, but a small fraction (2.0%) was donated to the CBG from external collections dating back to 1981. The primary techniques employed for collection were Malaise traps (N = 1,096,898), sweep netting (N = 87,910), flight-intercept traps (N = 69,162), pan traps (N = 67,225), pitfall traps (N = 43,771), ultraviolet (UV) light collections (N = 24,501) and manual collecting (N = 19,334).

#### ‘National parks’ subset

With support from Parks Canada, invertebrate surveys were conducted in 43 National Parks and National Park Reserves during 2012–2014; two thirds of the specimens (N = 644,510) were collected using Townes-style Malaise traps^37–39^ as part of the Global Malaise Trap Program (GMP) – Canada (www.globalmalaise.org)^40^. Most parks were only sampled during one year, but 12 were sampled in two or more years (Fig. 2b). The length of the collecting season for each park spanned most of the insect flight period, but was also determined by weather conditions and, for remote locations, by accessibility of the traps for servicing (see Supplementary File 1). Malaise traps were typically serviced weekly by Centre for Biodiversity Genomics (CBG) or Parks Canada staff by replacement of the sampling bottle with a bottle containing fresh preservative.

Additional specimens in the ‘National Parks’ subset (N = 300,828) were collected as part of the CBG’s Standardized Sampling Program. It ran from 2012–2014 in 23 National Parks, National Park Reserves, and National Urban Parks and employed a standard set of sampling methods that targeted a wider diversity of invertebrate fauna than Malaise traps^40^. In each Standardized Sampling (SS) locality, three representative sites were chosen based on a variety of biotic and abiotic factors, such as habitat type, vegetation, and elevation. The protocol was implemented over a one-week period and involved the deployment of a standard array of traps at each site: 1–2 Malaise traps, 1 flight-intercept trap, 10 pan traps, and 10–20 pitfall traps. In addition, 1–3 substrate samples were taken for Berlese funnel extraction, and a total of 60 min of sweep netting was performed over the week.

The remaining 56,832 (5.7%) specimens were obtained through opportunistic collecting in terrestrial, freshwater, and marine habitats using UV lights, dip nets, plankton nets, sieves, aspirators, mustard extraction, and freehand collecting.

#### ‘Other localities’ subset

The remaining 497,833 specimens in this subset were obtained through various methods and collection programs:

- the largest proportion of this subset (N = 166,835) was obtained through Malaise trapping at 35 additional protected areas (not national parks) for GMP – Canada^41^, including sites in proximity to ports in Vancouver, Montreal, Toronto, and Halifax (N = 71,747).
- an educational program – the School Malaise Trap Program (SMTP^42^) – deployed Malaise traps on school grounds across Canada, contributing a significant number of specimens (N = 93,378).
- all taxa biodiversity inventory (ATBI) and bioblitz^43^ activities combined to provide a large number of specimens (N = 83,277) using a diverse repertoire of methods. These included an ATBI at Churchill, MB from 2006 to 2009 (N = 41,449)^44–47^; a long-term inventory of the *rare* Charitable Research Reserve in Cambridge, ON (N = 41,608)^48^; and bioblitz^43^ events involving the Ontario BioBlitz (www.ontariobioblitz.ca) and Bioblitz Canada (www.bioblitzcanada.ca) programs.
- Other collections (N = 154,343) were likewise made with a variety of techniques, including our SS technique at protected areas (N = 22,943)

A summary of the collection method(s) used for each program is provided in Supplementary File 2. The collection method for each specimen is included in the data resources, as well as information on trap type, weather conditions, habitat, and any deviations from the normal collection protocol when this information is available. These programs covered 39 protected areas, including provincial parks, municipal parks, conservation reserves, ecological reserves, research reserves, and Nature Conservancy of Canada properties. Adding the collections in 43 national parks, a total of 1,132,347 occurrence records were derived from 82 protected areas across Canada.

### Specimen processing and DNA barcode analysis

The CBG has an efficient workflow for collecting, sorting, processing, and DNA barcoding specimens for reference library construction. As detailed protocols are outlined in other publications^49,50^, only summary details are provided here (Fig. 1).

Following collection, and prior to sorting, bulk samples and specimens were stored in −20 °C freezers, remaining in or transferred to 95% ethanol. All specimens from a trap sample or collection event were prepared for DNA barcoding, except in those cases where initial inspection suggested the presence of a very large number of specimens of a particular species. In these cases, 5 to 95 representatives of each morphospecies were prepared for sequence analysis and excess specimens were retained in ethanol at −20 °C. Larger specimens were pinned and one leg was removed for DNA extraction; smaller specimens were placed directly into 95% ethanol in either a) a sample tube rack, where a leg was later tissue-sampled for DNA extraction or b) a microplate, where the entire specimen was used for DNA extraction with an added step of recovering exoskeletal remains after non-destructive lysis and DNA extraction (‘voucher recovery’^51^).

Subsequent barcode analysis was performed following standard methods^50,52^; the stages include tissue lysis, DNA extraction, PCR amplification of the 658 base pair (bp) fragment of the cytochrome *c* oxidase subunit I (COI) gene, cycle sequencing, and subsequent Sanger sequence analysis. The resultant sequences, as well as electropherograms, and primer details for all specimens were uploaded to BOLD.

### Barcode Index Numbers

For all sequences uploaded to BOLD, the records were assigned to operational taxonomic units called Barcode Index Numbers (BINs) by the Refined Single Linkage (RESL) algorithm implemented on BOLD^53^. Individual records are either assigned to an existing BIN or found a new BIN, but they only enter the RESL analysis if they meet the following criteria: greater than 300 bp coverage of the barcode region, less than 1% ambiguous bases, and no stop codon or contamination of the sequence. For inclusion into an existing BIN, sequence records must include >300 bp of the barcode region (between positions 70 and 700 of the BOLD alignment) while records that establish a new BIN must include >500 bp of the barcode region. The RESL algorithm runs monthly on all qualifying barcode sequences in BOLD – which currently contains 7.7 million animal specimen records and 0.66 million BINs (November 2019). BIN designations and assignments generated by RESL on BOLD are accessible for independent validation through the ‘BIN pages’ that aggregate the specimen and sequence information of its members (e.g. the eastern yellowjacket wasp, *Vespula maculifrons* (Buysson): 10.5883/BOLD:AAD5593).

### Taxonomic assignment

Prior to processing, most specimens were identified to an order level based on morphology. After processing, each record went through a taxonomic assignment and verification workflow (see Supplementary File 3). Following a record’s assignment to a BIN, if that BIN contained specimens identified to a single family, genus or species, it received this identification. In cases of taxonomic discordance, the identification was applied above the level of disagreement. For example, if a BIN containing two members had one specimen assigned to genus A and the other to genus B, but both belonged to family C, the specimen would only be identified to the family level.

For specimens without a BIN assignment or where the taxonomy associated with the BIN was only to a family level, specimen sequences were compared to the complete reference library on BOLD using its Identification (BOLD-ID) Engine (available at http://v4.boldsystems.org/index.php/IDS_OpenIdEngine). A list of the top 99 sequence matches for each specimen was returned, and the taxonomy was applied where present and without discordance (as in BIN taxonomy assignment described above). Species-level identifications were assigned at ≥98% sequence similarity, genus-level identifications at ≥95% similarity, and family-level identifications at ≥90% similarity.

Specimens still lacking an identification at the family level were placed into a Neighbor-Joining tree of identified records in the same order, constructed on BOLD (see Supplementary File 4 for an example). If an unnamed specimen fell within a distinct haplogroup cluster, the lowest taxonomic level of agreement was applied to the specimen. If this approach was also unsuccessful, specimens were identified morphologically where possible, either by in-house experts or through loans to taxonomic specialists (e.g. Canadian National Collection of Insects, Arachnids, and Nematodes; Smithsonian Institution’s National Museum of Natural History; see Acknowledgments for key taxonomic specialists).

### Specimen, DNA, and image storage

All voucher specimens in the dataset were archived in a secure, microclimate-controlled Specimen Archive (BIOUG). All specimen provenance data, timing of processing, and storage locator information were digitized in a custom-designed institutional database (see Technical Validation below) to allow the efficient pre-laboratory processing, data submission, archival storage, and retrieval of specimens. All vouchers are available for loan for further research, and the data are accessible in various data portals (see Data Records below).

The DNA extracts produced during barcode analysis are stored within a DNA Archive, either in −80 °C freezers or dried in a trehalose or PVA-based cryoprotectant^54^ and held in −20 °C freezers. Information on these DNA extracts is stored in a MS Access database. Tracking of the DNA extracts through the DNA barcoding analytical steps was also captured by a custom-built PostgreSQL-based Laboratory Information Management System (BOLD-LIMS). The data necessary for the preparation of the specimen core and GGBN extension files were exported from the DNA Archive database and BOLD (see Data Records below).

Representatives of each BIN were photographed to build a digital image library to aid taxonomic validation. Specimens were photographed at high resolution and the images were made accessible through both the specimen and BIN pages on BOLD under Creative Commons No Rights Reserved (CC0 1.0) license.

## Data Records

### Records summary

Although the specimens were sourced from localities spanning ~4500 km in latitude and ~7000 km in longitude, sampling coverage was strongest in southern Canada (Fig. 2a). Sampling coverage varied between 13 provinces and territories more than 20-fold, with N = 13,225 (0.9%) for Nunavut versus N = 425,049 (28.3%) for Ontario. Most of the specimens (~98%) were from terrestrial habitats followed by freshwater (~1.5%) and marine (~0.5%) environments.

Most specimens associated with this data release are available for loan or further study in the Centre for Biodiversity Genomics Collection (BIOUG). A small percentage (2.3%) of specimens were damaged or lost during processing but, in nearly all cases, other representatives of that BIN were recovered. In total, 210,585 (14.0%) specimens were photographed and these images can be accessed on both the individual specimen and BIN pages. Most BINs (N = 58,126; 90.5%) in the data release are represented by an image of at least one voucher. When paired with Neighbor-Joining (NJ) trees, these images are critical for taxonomic validation and identification refinement (see Supplementary Files 4 and 5 for a NJ tree and associated images for one group of Canadian net-winged insects). The image library may also be useful as a training dataset for machine learning algorithms designed for specimen identification utilizing images (e.g.^55^).

This data release is taxonomically extensive as it includes representatives for 14 phyla, 43 classes, 163 orders, 1123 families, and 6186 genera. A very high proportion of the specimens have taxonomic assignments at the family (99.9%) and genus (69.5%) levels, but fewer (N = 571,902; 38.1%) could be assigned to a species (Table 1). Of the 1,500,003 specimens included in the resource, 1,457,334 (97.2%) were either placed into an established BIN on BOLD or founded a new one, for a total of 64,264 BINs. As a proxy for species, this BIN total represents a substantial gain for the Canadian species inventory. The last thorough compilation for all invertebrates^56,57^ indicated only 41,941 Canadian species and an estimated fauna of 78,821 species. Similarly, the more recent compilation of all terrestrial invertebrates by Langor^58^ assembled 44,100 described species with 27,000–42,600 remaining undiscovered and/or undescribed. Flies (Diptera) dominate both specimens (N = 875,215; 58.3%) and BINs (N = 27,525; 42.8%) in the current reference library, followed by bees, wasps, ants and allies (Hymenoptera) and moths and butterflies (Lepidoptera) (Table 1). The ‘Other Localities’ subset included 26.9% more families although it included half as many specimens as the Parks dataset. Taxonomic resolution (measured at the species level) also varied slightly between the subsets with the ‘National Parks’ subset at 35% identified to a species versus 44% for the ‘Other Localities’ subset. This variation in resolution is apparent between taxonomic categories as well; just 5–16% of mites and ticks (Acari) have a species assignment versus 99–100% for spiders (Araneae) and 82–87% for moths and butterflies (Lepidoptera).

**Table 1:** Summary data by major taxon represented within the dataset.

Subset Taxon	Specimens	BINs	# of Families	# of Named Species	Specimens to Family (%)	Specimens to Species (%)
**A. ‘National Parks’ subset**	1,002,170	49,501	818	10,563	100	35
Araneae	26,440	1,136	34	808	100	100
Acari	34,776	3,449	155	102	99	5
Collembola	30,723	781	16	54	100	45
Coleoptera	37,013	2,872	89	1,828	100	78
Diptera	616,492	23,330	101	2,593	100	28
Hemiptera	47,233	1,809	59	907	100	58
Hymenoptera	129,469	11,372	62	1,173	100	17
Lepidoptera	49,967	3,003	71	2,233	100	82
Other insects	24,964	1,195	95	644	100	59
Other invertebrates	5,093	554	137	221	99	70
**B. ‘Other Localities’ subset**	497,833	36,094	1,038	10,548	100	44
Araneae	14,414	972	34	788	100	99
Acari	22,221	3,127	160	153	99	16
Collembola	14,094	562	18	78	100	61
Coleoptera	18,551	2,109	84	1,433	100	77
Diptera	258,723	13,442	100	2,289	100	35
Hemiptera	25,672	1,553	63	819	100	58
Hymenoptera	73,992	8,605	59	1,207	100	22
Lepidoptera	41,289	2,943	68	2,402	100	87
Other insects	19,452	1,242	110	685	100	76
Other invertebrates	9,425	1,539	342	694	98	63
**Total**	**1,500,003**	**64,332**	**1,123**	**14,129**	**100**	**35**

A closer examination of the ‘National Parks’ subset reveals the recovery rate and overall complexity of the barcode-based workflow. In total, 1,148,787 specimens were processed from collecting events in these sites, but just 1,002,170 (87.2%) qualified for inclusion in the data release for four reasons. Firstly, 132,933 (11.6%) specimens were not successfully sequenced, with the order Hymenoptera comprising the largest proportion of failures (N = 46,103 failed specimens; recovery rate = 73.7%), followed by Diptera (N = 34,161; 94.8%), Acari (N = 17,189; 66.9%), and Hemiptera (N = 14,203; 76.9%). Secondly, ten sequence records contained stop codons, indicating that a pseudogene was likely sequenced instead of the COI barcode region; their low incidence (0.001%) indicates that nuclear mitochondrial pseudogenes (NUMTs; see^59^) rarely complicate the recovery of COI through Sanger sequencing, likely because the copy number of NUMTs is far less. Thirdly, 4,799 were flagged as possible contaminations or misidentifications. Fourthly, 6,100 specimens were excluded because their sequence was either <300 bp, had >1% ambiguous bp in the barcode fragment, or they lacked both a BIN and a family assignment. And lastly, as part of the taxonomic assignment workflow, 2,737 specimens were permanently transferred to other institutions so their vouchers are unavailable at the CBG.

**Fig. 4:**
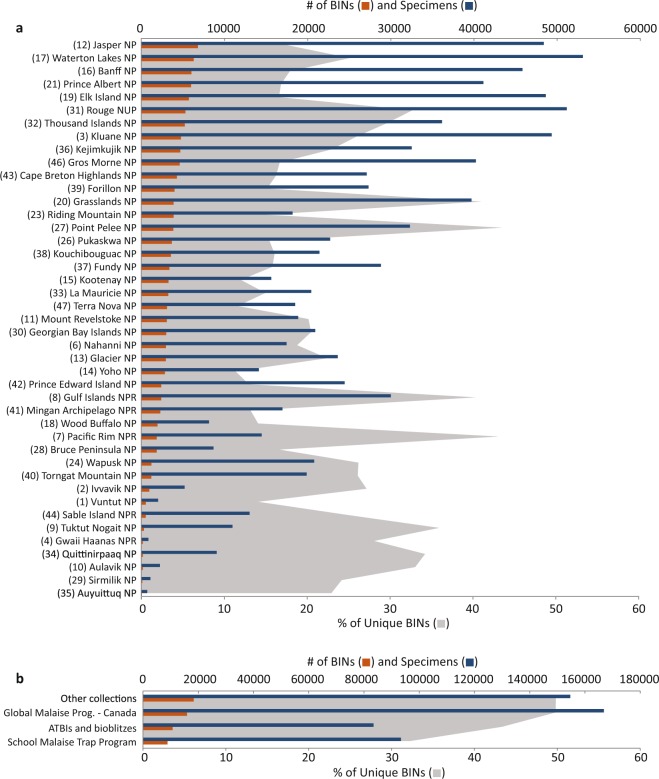
Specimen and BIN summaries for the two subsets in this data release. (**a**) ‘National Parks’ subset. Numbers correspond to those in Fig. 2 ranked by BIN count, and (**b**) ‘Other Localities’ subset. Numbers in parentheses correspond to Parks in Fig. 2. ‘Unique BINs’ refer to BINs collected only in that specific national park or collecting program.

Because collecting efforts in the national parks varied in frequency and length (Fig. 2b, Supplementary File 1), there was considerable variation in the number of BINs and specimens captured per park (Online-only Table 1, Fig. 4). Values ranged from a low of 77 BINs and 715 specimens at Auyuittuq National Park to 6,806 BINs and 48,405 specimens at Jasper National Park, with an average of 2,988 BINs and 23,3017 specimens per park. By comparison, in the ‘Other Localities’ subset, GMP - Canada captured 15,879 BINs and 166,835 specimens, SMTP captured 8,878 BINs and 93,378 specimens, and the combination of ATBIs and bioblitzes captured 10,721 BINs and 83,277 specimens. The sampling methods employed at each national park differed in some cases as well, further contributing to the disparity. As expected, these sampling methods each captured a differing subset of the local fauna, but in combination, they led to more comprehensive collections (Supplementary File 6).

### Records access

The specimen and sequence data for all 1,500,003 records are available on BOLD in public datasets (see list in Online-only Table 1, where specimens are grouped by national park and major collection programs; Fig. 3). The record for each specimen includes its date and locality of collection, its taxonomic assignment, and voucher specimen details. The record also includes trace files, quality scores, nucleotide sequence for the COI barcodes, and corresponding GenBank accession numbers. Condensed versions of the ‘National Parks’ and ‘Other Localities’ subsets, including full lists of GenBank accessions, are available in^60^. As noted earlier, 210,585 (14.0%) of the records possess a photograph of the specimen, all with the Creative Commons No Rights Reserved (CC0 1.0) license. Each specimen record has been publicly released and is searchable in the Public Data Portal on BOLD (www.boldsystems.org/index.php/Public_BINSearch) or downloadable by utilizing BOLD’s API (www.boldsystems.org/index.php/resources/api). Additionally, BOLD users can log in and search for any specimen(s) from the BOLD Workbench (http://www.boldsystems.org/index.php/Login/page). BOLD’s various methods of delivering the data permit a wide range of queries and subsequent analyses (BOLD data are available as a tab separated values file in^60^.

All sequences in this data release have been submitted to GenBank. A full list of GenBank Accessions for the ‘National Parks’ and ‘Other Localities’ subsets are available in^60^. From the GenBank homepage (https://www.ncbi.nlm.nih.gov/genbank/), accessions can be searched as a comma-separated list. The entire dataset can be accessed through the NCBI’s BioProject PRJNA472144 (www.ncbi.nlm.nih.gov/bioproject/472144)^61^.

After final validation, specimen data were uploaded to the Global Biodiversity Information Facility (GBIF; http://www.gbif.org) as a Darwin Core Archive^62^. The data are available from the University of Guelph’s installation of the Integrated Publishing Toolkit^63^ and webserver (https://ipt.uoguelph.ca/ipt/resource?r=cbg_canadian_specimens&v=1.4). The registered occurrence dataset can also be accessed directly from GBIF’s web portal (10.15468/mbwnw9), where it is available under a CC0 1.0 license^64^. This release on GBIF extends exposure for the occurrence data and supports the Convention on Biological Diversity (CBD) by providing a dataset useful for its assessments and indicators^65^, such as the Intergovernmental Science-Policy Platform on Biodiversity and Ecosystem Services (IPBES).

The DNA extracts derived from the 1,500,003 barcoded specimens are held in the DNA Archive at the CBG, either within −80 °C freezers or dried in a trehalose-based preservative and held in −20 °C freezers. The specimen data and DNA storage information were submitted to the Global Genome Biodiversity Network (GGBN) Data Portal^66^ following the GGBN Data Standard^67^. The upload of CBG’s DNA extract data added 566 families and 4,287 genera to GGBN, increasing the number of families and genera by 22% and 30%, respectively (based on an API download of Animalia records from GGBN in May 2019). The data are accessible – and the DNA extracts can be requested on a cost-recovery basis through the GGBN portal (http://www.ggbn.org/ggbn_portal/search/result?institution=BIOUG%2C+Guelph) or University of Guelph’s IPT (https://ipt.uoguelph.ca/ipt/resource?r=public_data&v=1.8).

## Technical Validation

### Inclusion in data release

Following taxonomic and sequence curation, specimens were required to pass one of two criteria before inclusion in the release dataset. First, specimens assigned to a BIN by the RESL algorithm (see Methods) were included. Second, if a specimen did not receive a BIN assignment, it was included in the dataset as long as its sequence was at least 300 bp long with <1% ambiguous base pairs, and led at least to a family-level assignment. No specimen whose sequence record was contaminated, had a stop codon, or was flagged by a member of the BOLD community (see Taxonomic Validation below) was included in the dataset.

### Sample tracking

Using a custom-built collection information management system (CIMS), the specific location and storage medium for each specimen was captured at the time of its submission to the CBG’s collection archive. Unlike most natural history collections, specimens are arranged in order of processing to permit rapid submission of new specimens (up to 40,000 per week), to facilitate specimen retrieval (e.g. for photography), and to optimize the use of cabinet space. Because every specimen in the archive is databased, it is possible to query the CIMS (e.g. by a list of BINs, or a taxon for a particular geographical area) and quickly assemble all specimens required for an external loan or for examination by a visiting researcher.

### Taxonomic validation

Multiple curatorial efforts were undertaken to validate taxonomic assignments. Taxonomic conflicts within BINs were investigated and resolved where possible. This review often led to a persistent flag in BOLD stating that the record is contaminated or misidentified (which works much like a wiki – see^68^). The list of matches provided by the BOLD-ID Engine was checked for taxonomic discordances indicative of contaminated samples or misidentified specimens and corresponding data records were flagged. Neighbor-Joining (NJ) trees of similar taxa (at the order level) were constructed on BOLD to reveal unexpected placements of taxa; this also included evaluation of an image library paired to the tree to facilitate the recognition of specimens whose phenotype was incongruent with its taxonomic assignment (see Supplementary Files 4 and 5 for an example NJ tree and associated images for Canadian net-winged insects, Neuroptera).

All species-level identifications were validated against current nomenclature. The first validation pass included comparisons against national or regional checklists (e.g.^69^ for true bugs^70^ ; for beetles; and^71^ for moths and butterflies). Taxa that did not match with authoritative checklists were verified against online resources such as the Catalog of Life, WoRMs, ITIS, GBIF, or the World Spider Catalog. Remaining names were searched on a case-by-case basis in the taxonomic literature. Any synonyms or misspellings that were detected were corrected to the valid name.

### Sequence validation

DNA sequences submitted to BOLD are first translated into amino acids and are then compared against a Hidden Markov Model of the COI protein. This pre-screening identifies gaps that provoke a frameshift or a stop codon, and other sequencing or editing errors. Sequences found to possess potential errors were manually re-edited or re-assembled from chromatogram trace files in CodonCode Aligner which often enabled the correction of errors made during the initial sequence editing. Sequences with confirmed gaps leading to frameshifts were excluded from the dataset. After initial submission to NCBI, staff at GenBank would report any residual errors detected with their validation tools allowing their correction before final submission.

## Usage Notes

The DNA barcode reference library presented here, covering nearly 65,000 species of Canadian invertebrates, should have wide utility in supporting specimen identifications through barcoding and metabarcoding. Its primary use will undoubtedly derive from its capacity to assign unknown specimens and samples to a taxon. This step is key in producing accurate and reproducible data in metabarcoding studies^72,73^. The present DNA barcode reference library should also aid in quality control and validation for whole genome analysis by detecting misidentified samples and revealing cases of contamination (e.g.^74^). While the library will be most useful for work in Canada, a third of the species found in the Nearctic occurs in Canada, and about 5% of the Holarctic fauna, meaning the library will have utility across the Holarctic region. In fact, given its taxonomic breadth – 14 phyla, 43 classes, 163 orders, and 1123 families – it should be useful for studies worldwide, particularly for terrestrial invertebrates. It should also be valuable as a model for library construction in other countries and for other environments (e.g. soils, oceans), in Canada and elsewhere. In all applications, the accessibility of the library in various repositories^60,61,64^, paired with the ongoing curation and refinement of taxonomic assignments by the biodiversity science community, further ensures its value will increase through time.

## Supplementary information

Supplementary Information.

## Data Availability

The Barcode of Life Data System (BOLD; www.boldsystems.org)^8^ was used as the primary workbench for creating, storing, analyzing, and validating the specimen and sequence records and the associated data resources^48^. The BOLD platform has a private, password-protected workbench for the steps from specimen data entry to data validation (see details in Data Records), and a public data portal for the release of data in various formats. The latter is accessible through an API (http://www.boldsystems.org/index.php/resources/api?type=webservices) that can also be controlled through R^75^ with the package ‘bold’^76^.

## Online-only Table

**Online-only Table 1:** Summary data and BOLD datasets for each park or program in the ‘National Parks’ and ‘Other Localities’ subsets. Park numbers correspond to those in Fig. 2.

No.	National Park (+BOLD dataset)	Province/Territory	Park Area (km²)	Sampling Year	Sampling Type	No. of Sampling Events	No. of Specimens
**A**. **‘National Parks’ subset**							
1	Vuntut National Park DS-BBVNP1	YT	4,345	2014	Malaise	2	2,007
2	Ivvavik National Park DS-BBINP1	YT	10,168	2014	Malaise	8	5,217
3	Kluane National Park Reserve DS-BBKLNP1	YT	22,013	2014	Malaise/SS/General	56	49,337
4	Gwaii Haanas National Park Reserve DS-BBGHNP1	BC	1,470	2014	Malaise	11	854
5	Nááts’ihch’oh National Park Reserve N/A	NT	4,850	N/A	N/A	N/A	N/A
6	Nahanni National Park Reserve DS-BBNNP1	NT	30,050	2014	Malaise	7	17,464
7	Pacific Rim National Park Reserve DATASET-BBPRNP1	BC	511	2012, 2014	Malaise/SS/General	252	14,463
8	Gulf Islands National Park Reserve DS-BBGINP1	BC	33	2012, 2014	Malaise/SS/General	90	30,005
9	Tuktut Nogait National Park DS-BBTNNP1	NT	18,100	2014	Malaise	2	10,971
10	Aulavik National Park DS-BBALNP1	NT	12,200	2014	Malaise	2	2,225
11	Mount Revelstoke National Park DATASET-BBMRNP1	BC	260	2012, 2014	Malaise/General	126	18,857
12	Jasper National Park DATASET-BBJNP1	AB	10,878	2012	Malaise/SS/General	483	48,405
13	Glacier National Park DATASET-BBGCNP1	BC	1,349	2012, 2014	Malaise/SS/General	131	23,617
14	Yoho National Park DATASET-BBYNP1	BC	1,313	2014	Malaise	173	14,143
15	Kootenay National Park DATASET-BBKTNP1	BC	1,406	2014	Malaise/SS/General	171	15,622
16	Banff National Park DATASET-BBBNP1	AB	6,641	2012, 2014	Malaise/SS/General	303	45,842
17	Waterton Lakes National Park DATASET-BBWLNP1	AB	505	2012	Malaise/SS/General	445	53,095
18	Wood Buffalo National Park DS-BBWBNP1	AB/NT	44,807	2012	Malaise/General	26	8,137
19	Elk Island National Park DATASET-BBEINP1	AB	194	2012	Malaise/SS/General	267	48,640
20	Grasslands National Park DATASET-BBGNP1	SK	907	2012, 2014	Malaise/SS/General	99	39,703
21	Prince Albert National Park DATASET-BBPANP1	SK	3,874	2012	Malaise/SS/General	395	41,142
22	Qausuittuq National Park	NU	11,000	N/A	N/A	N/A	N/A
23	Riding Mountain National Park DATASET-BBRMNP1	MB	2,969	2012	Malaise	312	18,195
24	Wapusk National Park DS-BBWNP1	MB	11,475	2014	Malaise	8	20,793
25	Ukkusiksalik National Park N/A	NU	20,500	N/A	N/A	N/A	N/A
26	Pukaskwa National Park DATASET-BBPNP1	ON	1,878	2013	Malaise	188	22,715
27	Point Pelee National Park DATASET-BBPPNP1	ON	15	2012, 2014	Malaise/SS/General	582	32,288
28	Bruce Peninsula National Park DATASET-BBBPNP1	ON	154	2012, 2014	Malaise/SS/General	126	8,675
29	Sirmilik National Park DS-BBSNP1	NU	22,200	2014	Malaise	2	1,096
30	Georgian Bay Islands National Park DS-BBGBNP1	ON	13.5	2013, 2014	Malaise/SS/General	16	20,920
31	Rouge National Urban Park DS-BBRNUP1	ON	79	2013, 2014	Malaise/SS/General	352	51,162
32	Thousand Islands National Park DS-BBTINP1	ON	24.4	2012, 2014	Malaise/SS/General	31	36,165
33	La Mauricie National Park DS-BBLMNP1	QC	536	2013	Malaise	19	20,433
34	Quttinirpaaq National Park DS-BBQNP1	NU	37,775	2014	Malaise	10	9,065
35	Auyuittuq National Park DS-BBAYNP1	NU	19,089	2014	Malaise	1	715
36	Kejimkujik National Park DATASET-BBKJNP1	NS	404	2013	Malaise/SS/General	209	32,506
37	Fundy National Park DATASET-BBFNP1	NB	207	2013	Malaise/SS/General	169	28,815
38	Kouchibouguac National Park DATASET-BBKCNP1	NB	238	2013	Malaise/SS/General	183	21,420
39	Forillon National Park DS-BBFONP1	QC	244	2013	Malaise	21	27,319
40	Torngat Mountain National Park DS-BBTMNP1	NL	9,700	2013, 2014	Malaise	9	19,880
41	Mingan Archipelago National Park Reserve DS-BBMANP1	QC	151	2013	Malaise	17	16,978
42	Prince Edward Island National Park DS-BBPEINP1	PE	22	2013	Malaise/SS/General	69	24,443
43	Cape Breton Highlands National Park DATASET-BBCBNP1	NS	949	2013	Malaise/SS/General	273	27,103
44	Sable Island National Park Reserve DS-BBSINP1	NS	30	2014	Malaise	173	13,020
45	Mealy Mountains National Park Reserve N/A	NL	10,700	N/A	N/A	N/A	N/A
46	Gros Morne National Park DATASET-BBGMNP1	NL	1,805	2013	Malaise/SS/General	177	40,234
47	Terra Nova National Park DATASET-BBTNNP1	NL	400	2013	Malaise/SS/General	194	18,484
**B**. **‘Other Localities’ subset**							
—	Global Malaise Canada DS-GMPC1, DS-GMPC2	CAN	N/A	2012–2017	Malaise	151	166,835
—	School Malaise Trap Program DS-SMTPC	CAN	N/A	2013–2017	Malaise	407	93,378
—	ATBIs and bioblitzes DS-ATBIB	ON/MB	N/A	2006–2017	Malaise/SS/General	18,453	83,277
—	Other collections DS-OLOCC1, DS-OLOCC2	CAN	N/A	2006–2017	Malaise/SS/General	28,829	154,343
