# Supplementary material for: A reference library for Canadian invertebrates with 1.5 million barcodes, voucher specimens, and DNA samples
Source: Sci Data. 2019 Dec 6;6:308. doi: 10.1038/s41597-019-0320-2 (PMC6897906; doi:10.1038/s41597-019-0320-2)

Supplementary Information

Supplementary files to accompany deWaard et al. – 'A reference library for Canadian invertebrates with 1.5 million barcodes, voucher specimens, and DNA samples'.

Supplementary File 1. Duration of sampling from 2012-2014 as visualized by the number of Julian days of collecting for the 'National Parks' subset. ……………………………………………………………………………………2

Supplementary File 2. Breakdown of the methods used in the five major collection programs. ……………….3

Supplementary File 3. Taxonomic assignment workflow. ……………………………………………………………………..4

Supplementary File 4. Example BOLD Neighbor-Joining tree for Canadian net-winged insects (Neuroptera). ……5

Supplementary File 5. Example BOLD image library (associated with Supplementary File 4) for Canadian net-winged insects (Neuroptera). ……………………………………………………………………………………………………6

Supplementary File 6. Taxonomic breakdown for the eight major collection methods used in the 'National Parks' subset. ……11

Supplementary File 1. Duration of sampling from 2012-2014 as visualized by the number of Julian days of collecting for the 'National Parks' subset.

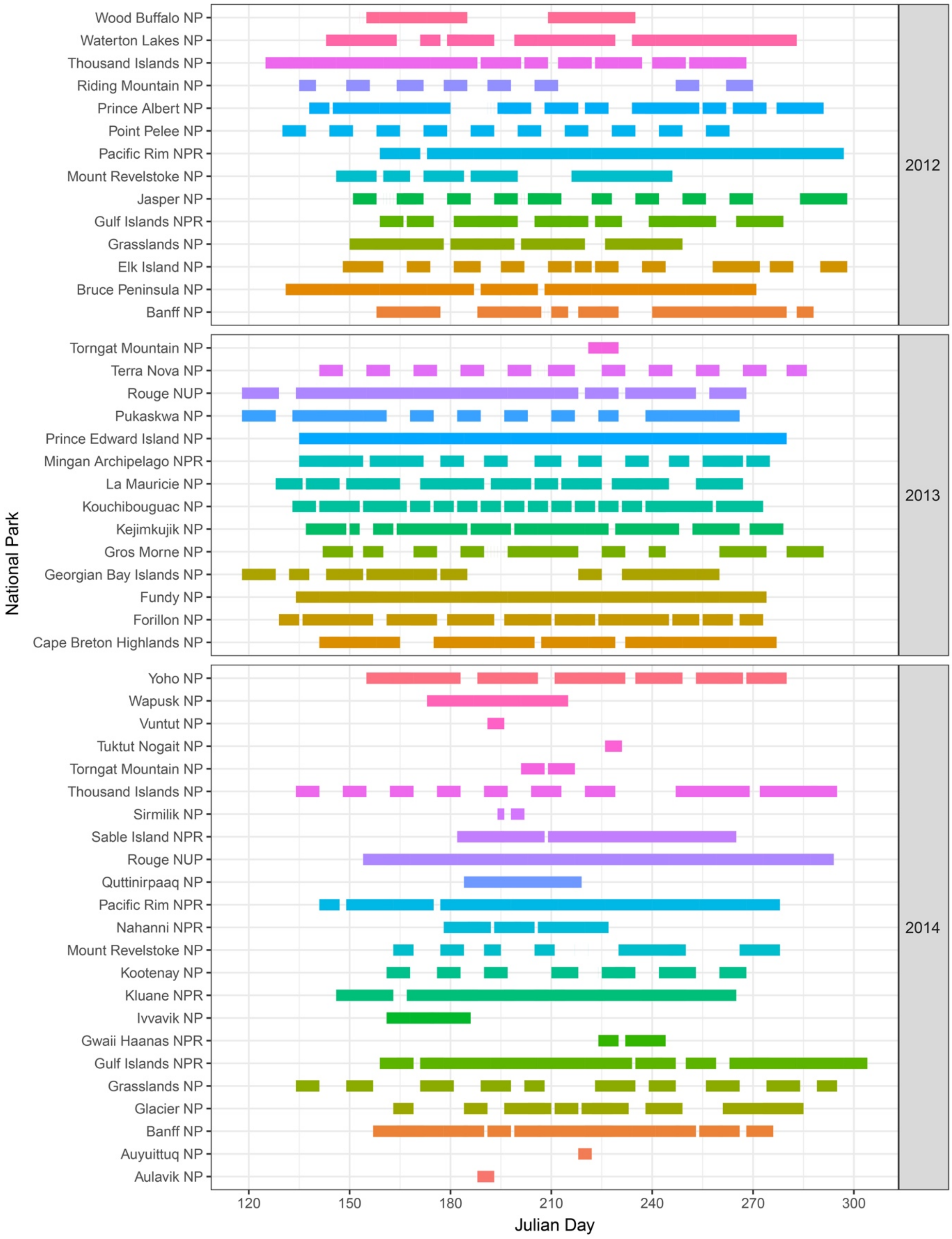

Supplementary File 2. Breakdown of the methods used in the five major collection programs.

	A. 'National Parks' subset			B. 'Other Localities' subset			
	**Global Malaise Program - Canada**	**Standardized Sampling Program**	**Other Collections**	**Global Malaise Program - Canada**	**School Malaise Trap Program**	**All taxa biodiversity inventories and bioblitzes**	**Other Collections**
Malaise Trap	x	x	x	x	x	x	x
Intercept Trap		x	x			x	x
Pan Trap		x	x			x	x
UV Light Trap			x			x	x
UV Light Sheet			x			x	x
Pitfall trap		x	x			x	x
Sweep Net		x	x			x	x
Free Hand			x			x	x
Berlese Funnel			x			x	x
UV Bucket Trap			x			x	
Dip net			x			x	x
Bottle Trap			x			x	x
Mustard Extraction			x				x
Sieve			x				x
Plankton Net			x			x	x

Supplementary File 3. Taxonomic assignment workflow.

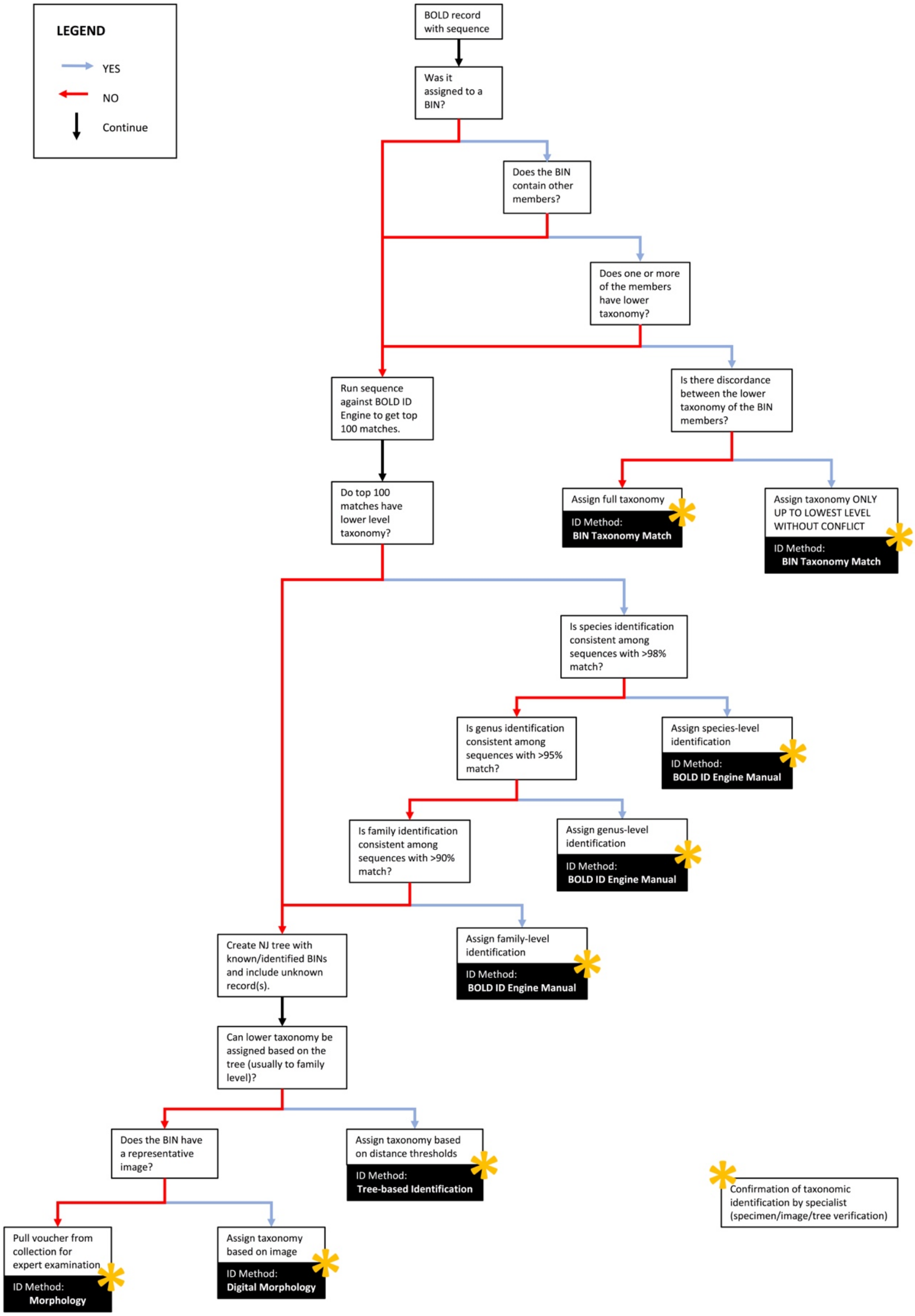

Supplementary File 4. Example BOLD Neighbor-Joining tree for Canadian net-winged insects (Neuroptera).

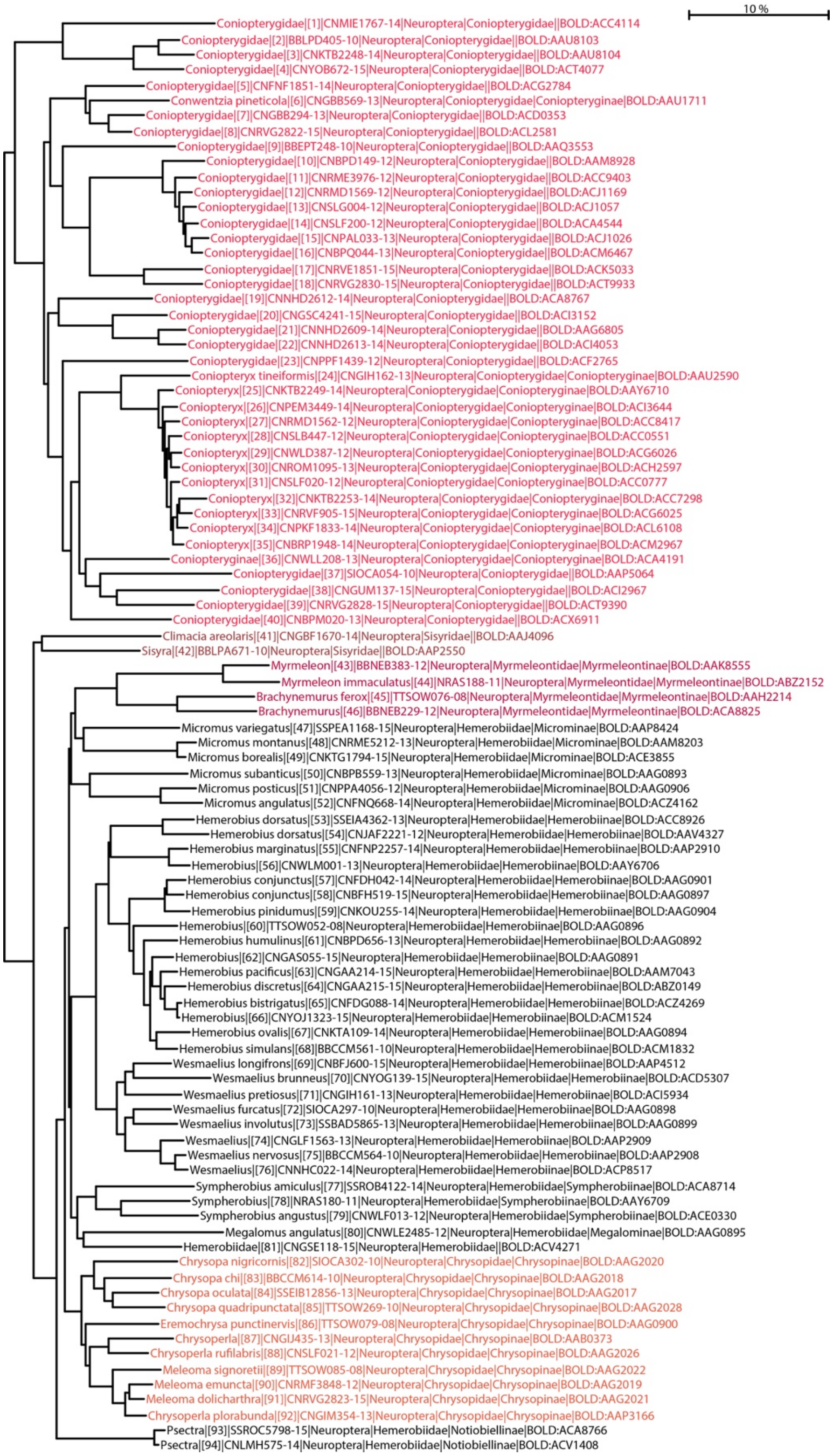

Supplementary File 5. Example BOLD image library (associated with Supplementary File 3) for Canadian net-winged insects (Neuroptera).

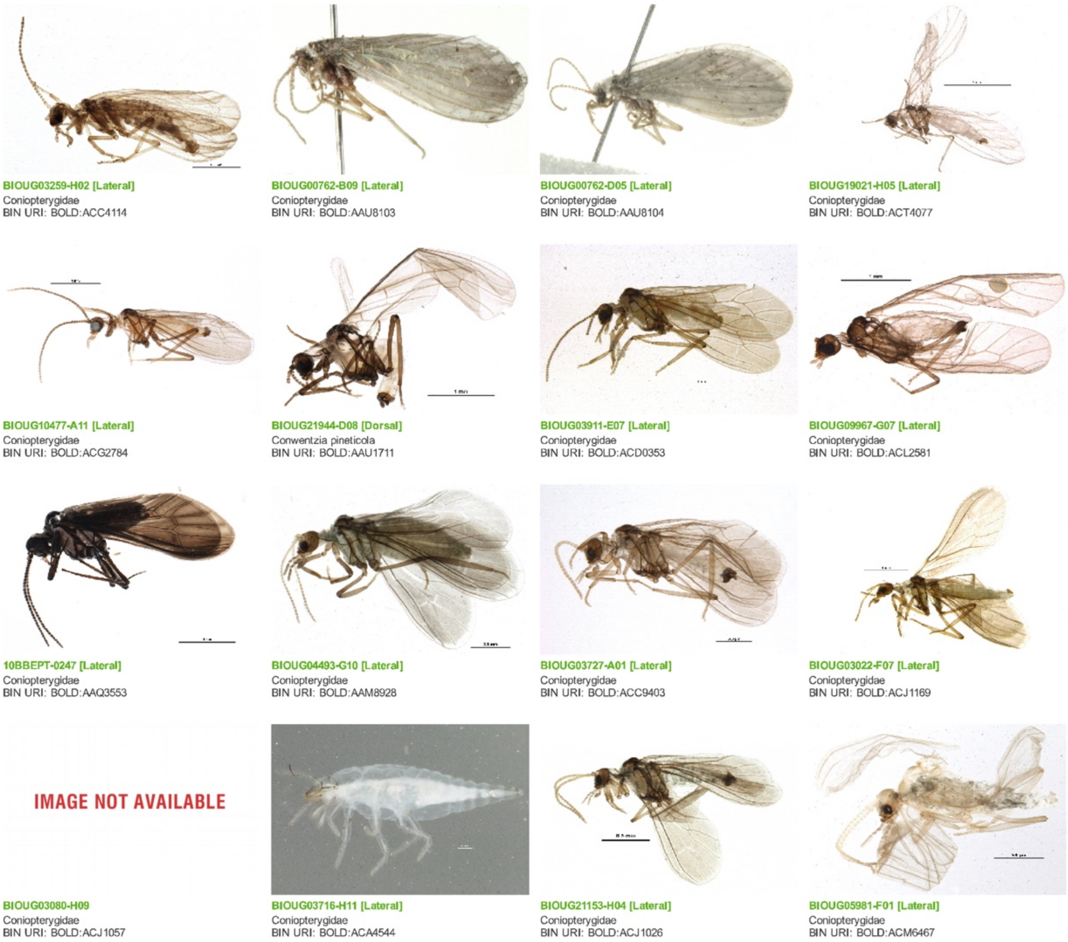

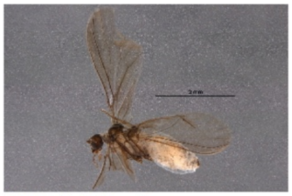

BIOUG07110-C06 [Lateral]
Coniopterygidae
BIN URI: BOLD:ACK5033

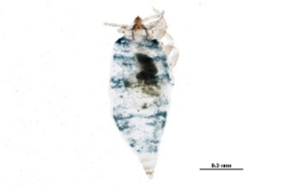

BIOUG20369-F05 [Dorsal]
Coniopterygidae
BIN URI: BOLD:ACT9933

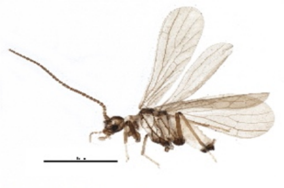

BIOUG17693-G11 [Lateral]
Coniopterygidae
BIN URI: BOLD:ACA8767

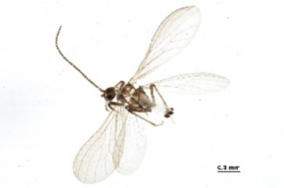

BIOUG07171-B08 [Lateral]
Coniopterygidae [skewed]
BIN URI: BOLD:ACI3152

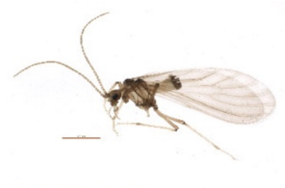

BIOUG03280-F10 [Lateral]
Coniopterygidae
BIN URI: BOLD:AAG6805

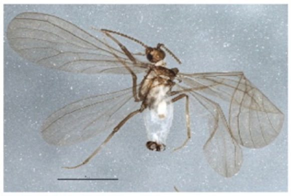

BIOUG06018-H10 [Dorsal]
Coniopterygidae
BIN URI: BOLD:ACI4053

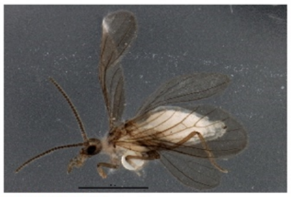

BIOUG03566-A09 [Lateral]
Coniopterygidae
BIN URI: BOLD:ACF2765

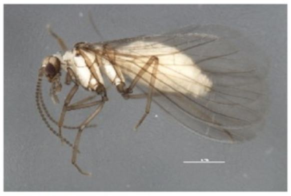

BIOUG00864-E03 [Lateral]
Coniopteryx tineiformis
BIN URI: BOLD:AAU2590

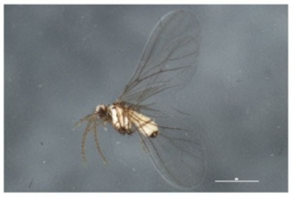

BIOUG00864-E04 [Lateral]
Coniopteryx
BIN URI: BOLD:AAY6710

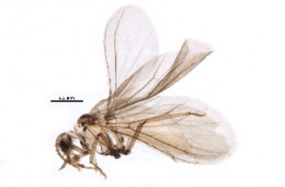

BIOUG11087-G10 [Lateral]
Coniopteryx
BIN URI: BOLD:ACI3644

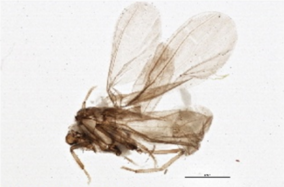

BIOUG03022-E12 [Lateral]
Coniopteryx
BIN URI: BOLD:ACC8417

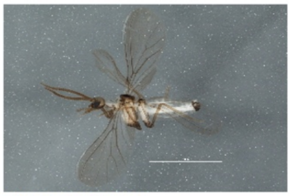

BIOUG02751-B10 [Lateral]
Coniopteryx
BIN URI: BOLD:ACC0551

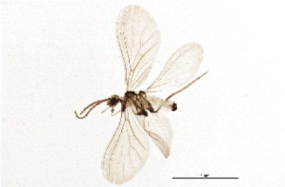

BIOUG03259-G04 [Lateral]
Coniopteryx
BIN URI: BOLD:ACG6026

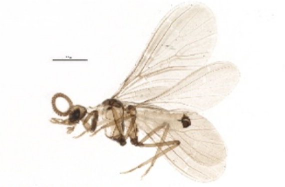

BIOUG06869-F09 [Lateral]
Coniopteryx
BIN URI: BOLD:ACH2597

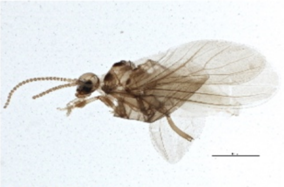

BIOUG04967-C05 [Lateral]
Coniopteryx
BIN URI: BOLD:ACC0777

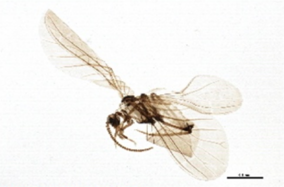

BIOUG03259-G06 [Lateral]
Coniopteryx
BIN URI: BOLD:ACC7298

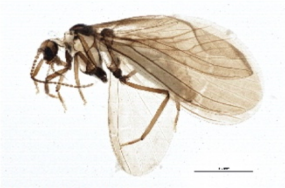

BIOUG03259-G07 [Lateral]
Coniopteryx
BIN URI: BOLD:ACG6025

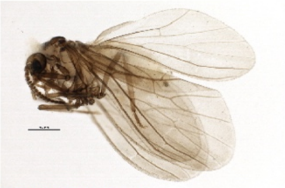

BIOUG10296-F02 [Lateral]
Coniopteryx
BIN URI: BOLD:ACL6108

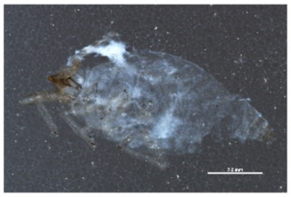

BIOUG11215-B08 [Lateral]
Coniopteryx
BIN URI: BOLD:ACM2967

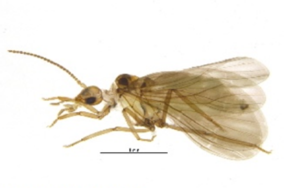

BIOUG03566-B01 [Lateral]
Coniopterygidae
BIN URI: BOLD:ACA4191

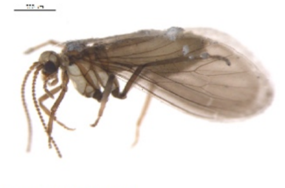

10BBSIO-0054 [Lateral]
Coniopterygidae
BIN URI: BOLD:AAP5064

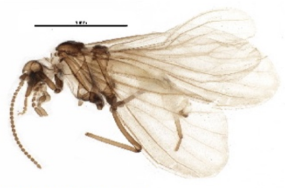

BIOUG06945-F06 [Lateral]
Coniopterygidae
BIN URI: BOLD:ACI2967

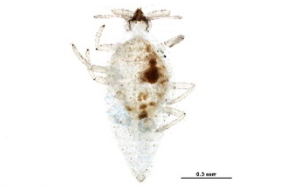

BIOUG20369-F03 [Dorsal]
Coniopterygidae
BIN URI: BOLD:ACT9390

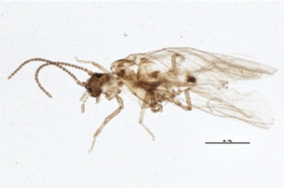

BIOUG05828-E10 [Lateral]
Coniopterygidae
BIN URI: BOLD:ACX6911

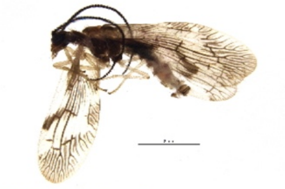
BIOUG10061-H03 [Lateral]
Climacia areolaris
BIN URI: BOLD:AAJ4096

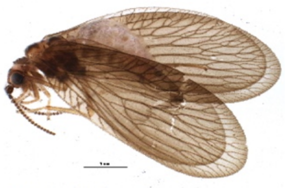
BIOUG08286-F11 [Lateral]
Sisyra
BIN URI: BOLD:AAP2550

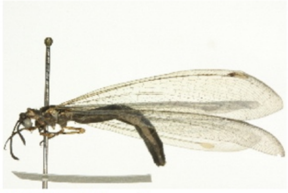
BIOUG02806-F02 [Lateral]
Myrmeleon
BIN URI: BOLD:AAK8555

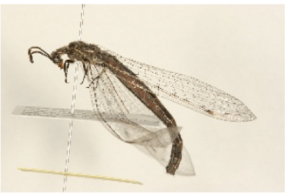
09BBNEU-0055 [Lateral]
Myrmeleon immaculatus
BIN URI: BOLD:ABZ2152

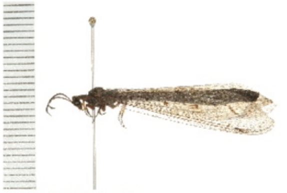
08BBNEU-076 [Lateral]
Brachynemurus ferox
BIN URI: BOLD:AAH2214

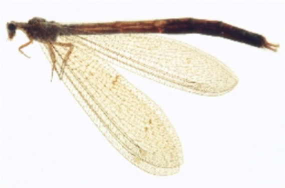
BIOUG02805-A01 [Lateral]
Brachynemurus
BIN URI: BOLD:ACA8825

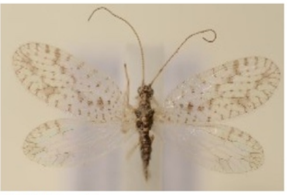
TR00040 [Dorsal]
Micromus variegatus
BIN URI: BOLD:AAP8424

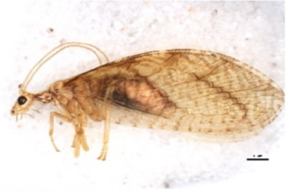
09BBNEU-0142 [Lateral]
Micromus montanus
BIN URI: BOLD:AAM8203

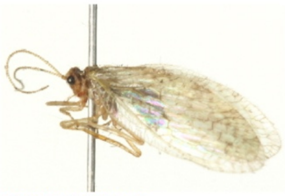
BIOUG00762-F11 [Lateral]
Micromus borealis
BIN URI: BOLD:ACE3855

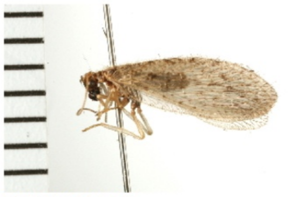
08BBNEU-013 [Lateral]
Micromus subanticus
BIN URI: BOLD:AAG0893

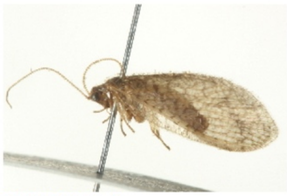
PHFLO-0106 [Lateral]
Micromus posticus
BIN URI: BOLD:AAG0906

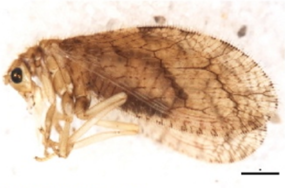
09BBNEU-0148 [Lateral]
Micromus angulatus
BIN URI: BOLD:ACZ4162

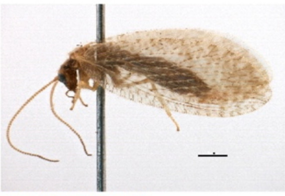
BIOUG10951-B11 [Lateral]
Hemerobius dorsatus
BIN URI: BOLD:ACC8926

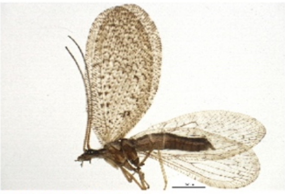
BIOUG03505-C06 [Lateral]
Hemerobius dorsatus
BIN URI: BOLD:AAV4327

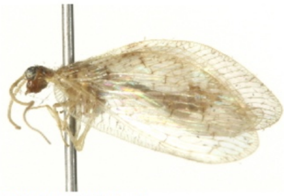
BIOUG00762-F03 [Lateral]
Hemerobius
BIN URI: BOLD:AAP2910

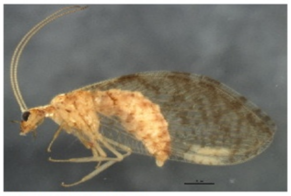
BIOUG00864-A06 [Lateral]
Hemerobius
BIN URI: BOLD:AAY6706

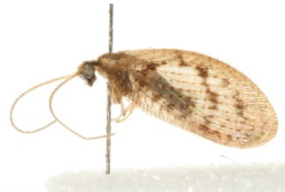
08BBNEU-088 [Lateral]
Hemerobius ovalis
BIN URI: BOLD:AAG0901

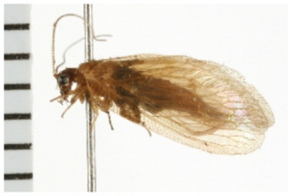
08BBNEU-063 [Lateral]
Hemerobius conjunctus
BIN URI: BOLD:AAG0897

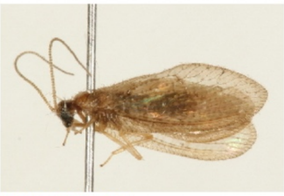
09BBNEU-0095 [Lateral]
Hemerobius pinidumus
BIN URI: BOLD:AAG0904

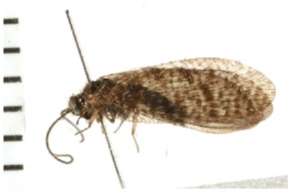
08BBNEU-052 [Lateral]
Hemerobius
BIN URI: BOLD:AAG0896

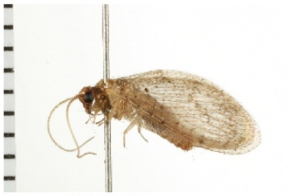
08BBNEU-005 [Lateral]
Hemerobius humulinus
BIN URI: BOLD:AAG0892

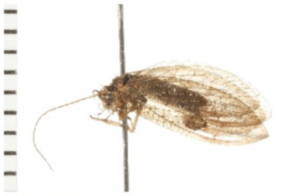
08BBNEU-018 [Lateral]
Hemerobius
BIN URI: BOLD:AAG0891

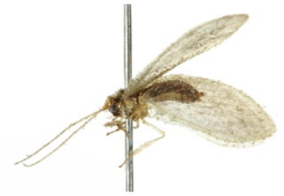
10BBNEU-0006 [Lateral]
Hemerobius
BIN URI: BOLD:AAM7043

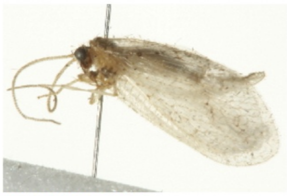
BIOUG00762-D07 [Lateral]
Hemerobius discretus
BIN URI: BOLD:ABZ0149

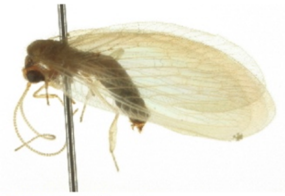

BIOUG02795-A09 [Lateral]
Hemerobius
BIN URI: BOLD:ACZ4269

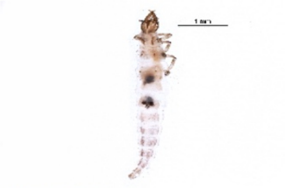

BIOUG19485-B06 [Larva]
Hemerobius
BIN URI: BOLD:ACM1524

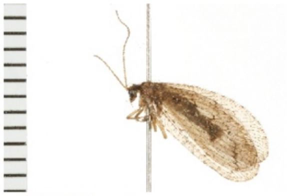

08BBNEU-021 [Lateral]
Hemerobius ovalis
BIN URI: BOLD:AAG0894

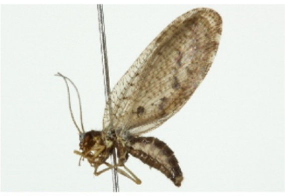

09BBNEU-0169 [Lateral]
Hemerobius simulans
BIN URI: BOLD:ACM1832

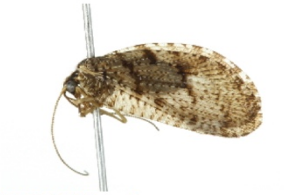

09BBNEU-0173 [Lateral]
Wesmaelius longifrons
BIN URI: BOLD:AAP4512

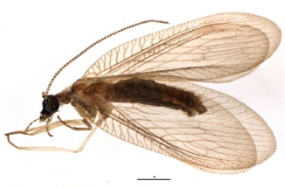

BIOUG05803-G04 [Lateral]
Wesmaelius brunneus
BIN URI: BOLD:ACD5307

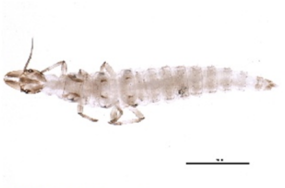

BIOUG16124-G03 [Larva]
Wesmaelius pretiosus
BIN URI: BOLD:ACI5934

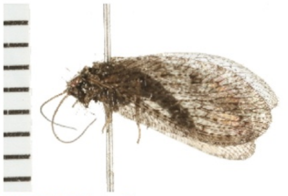

08BBNEU-066 [Lateral]
Wesmaelius furcatus
BIN URI: BOLD:AAG0898

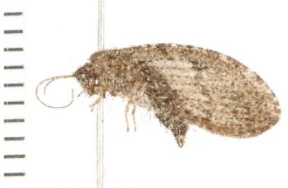

08BBNEU-071 [Lateral]
Wesmaelius involutus
BIN URI: BOLD:AAG0899

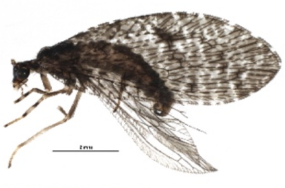

BIOUG20171-E09 [Lateral]
Wesmaelius
BIN URI: BOLD:AAP2909

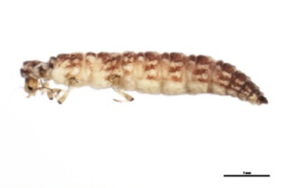

BIOUG00676-A12 [Lateral]
Wesmaelius nervosus
BIN URI: BOLD:AAP2908

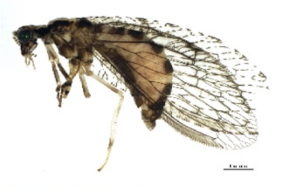

BIOUG16073-F03 [Lateral]
Wesmaelius
BIN URI: BOLD:ACP8517

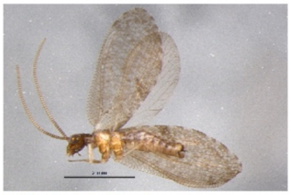

BIOUG02863-D02 [Lateral]
Sympherobius amiculus
BIN URI: BOLD:ACA8714

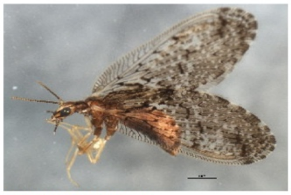

BIOUG00864-D03 [Lateral]
Sympherobius
BIN URI: BOLD:AAY6709

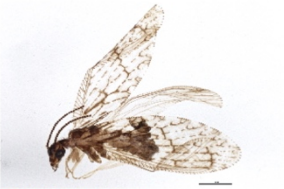

BIOUG03522-C11 [Lateral]
Sympherobius angustus
BIN URI: BOLD:ACE0330

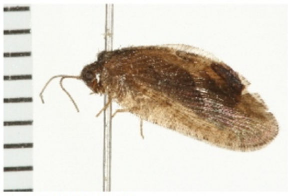

08BBNEU-050 [Lateral]
Megalomus angulatus
BIN URI: BOLD:AAG0895

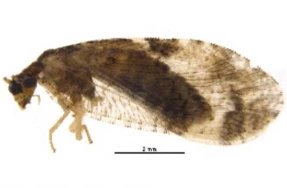

BIOUG21523-F03 [Lateral]
Hemerobiidae
BIN URI: BOLD:ACV4271

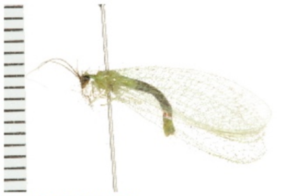

08BBNEU-057 [Lateral]
Chrysopa nigricornis
BIN URI: BOLD:AAG2020

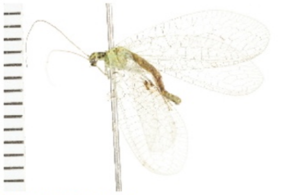

08BBNEU-053 [Lateral]
Chrysopa chi
BIN URI: BOLD:AAG2018

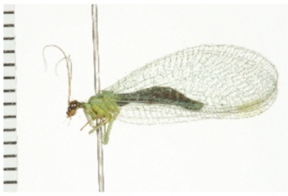

08BBNEU-033 [Lateral]
Chrysopa oculata
BIN URI: BOLD:AAG2017

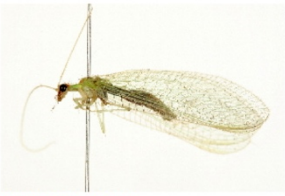

09BBNEU-0003 [Lateral]
Chrysopa quadripunctata
BIN URI: BOLD:AAG2028

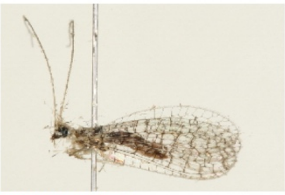

09BBNEU-0097 [Lateral]
Eremochrysa punctinervis
BIN URI: BOLD:AAG0900

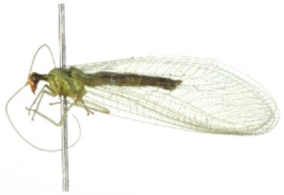

10BBNEU-0012 [Lateral]
Chrysoperla
BIN URI: BOLD:AAB0373

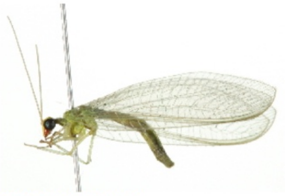

10BBNEU-0033 [Lateral]
Chrysoperla rufilabris
BIN URI: BOLD:AAG2026

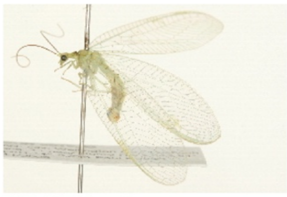

NEUR 0019.02 [Lateral]
Meleoma signoretii
BIN URI: BOLD:AAG2022

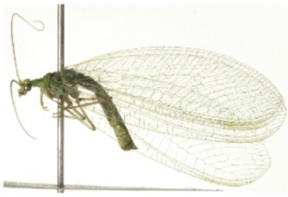

BIOUG00762-B01 [Lateral]
Meleoma emuncta
BIN URI: BOLD:AAG2019

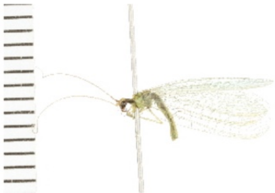

08BBNEU-073 [Lateral]
Meleoma dolicharthra
BIN URI: BOLD:AAG2021

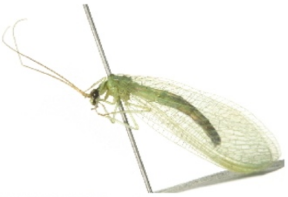

BIOUG00762-F12 [Lateral]
Chrysoperla plorabunda
BIN URI: BOLD:AAP3166

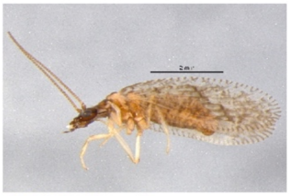

BIOUG02863-D11 [Lateral]
Psectra
BIN URI: BOLD:ACA8766

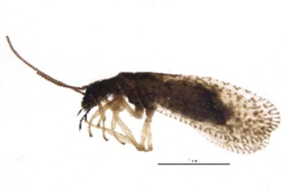

BIOUG11001-E06 [Lateral]
Psectra
BIN URI: BOLD:ACV1408

Supplementary File 6. Taxonomic breakdown for the eight major collection methods used in the 'National Parks' subset. Taxonomic breakdown is summarized for a) specimens and b) BINs captured.

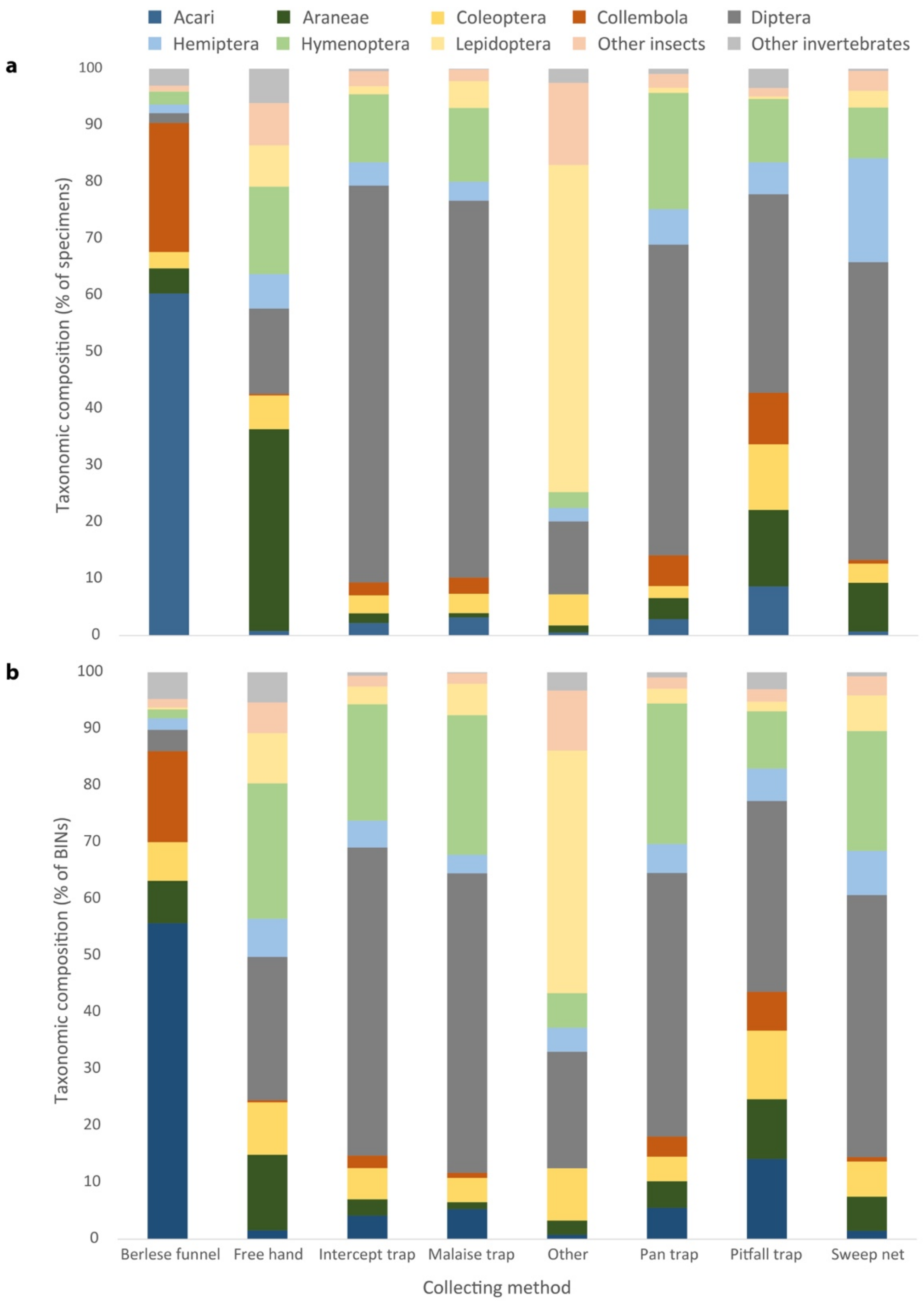

Supplement: Supplementary file 1 — Supplementary Information. [file 41597_2019_320_MOESM1_ESM.pdf]
